# The Impact of Marital Status and Employment Disruption on Food Insecurity Among a National Sample of Cancer Survivors

**DOI:** 10.7759/cureus.117334

**Published:** 2026-10-01

**Authors:** LaMarcus T Wingate, Faith Ogini, Rakchhya Uprety, Ruth Demessie, Salome Weaver, Folashade Onagbeso, Natasha R Brown, Martina Bakhoum, Nkem P Nonyel

**Affiliations:** 1 Department of Clinical and Administrative Pharmacy Sciences, Howard University College of Pharmacy, Washington, D.C., USA; 2 Department of Communication, Indiana University Northwest, Gary, IN, USA

**Keywords:** cancer survivors, employment disruption, financial toxicity, food insecurity, health disparities, marital status

## Abstract

Background: Emerging evidence suggests that financial toxicity may be associated with a greater likelihood of experiencing food insecurity in cancer survivors. Although marital status is an established correlate of food insecurity in the general population, its role among cancer survivors remains understudied. The objective of this study was to examine the association between the likelihood of food insecurity and employment disruption and marital status in a national sample of cancer survivors.

Methods: A cross-sectional study was conducted using data from the 2016-2017 panels of the Medical Expenditure Panel Survey (MEPS) Experiences with Cancer Survivorship Supplement. Marital status was categorized as married, formerly married, or never married, and employment status was categorized according to full employment following cancer diagnosis, no employment at time of diagnosis, or employment disruption. Weighted logistic regression models were constructed to evaluate the association between marital status, employment status, and food insecurity.

Results: Survivors experiencing employment disruption had higher odds of food insecurity compared with those who retained full employment (adjusted odds ratio (aOR) = 2.46; p = 0.005). Formerly married (aOR = 2.14; p = 0.003) and never-married survivors (aOR = 2.52; p = 0.014) also had higher odds of food insecurity compared with married survivors.

Conclusion: Cancer survivors who are unmarried and experience employment disruption have a higher likelihood of experiencing food insecurity.

## Introduction

There are nearly 19 million Americans living with a history of cancer [1]. After overcoming cancer, many individuals and families continue to face financial toxicity in the form of ongoing financial and economic burdens associated with the disease [2]. Costs related to medical treatment, such as medications, hospitalizations, and supportive care not covered by insurance, are one cause of financial toxicity [3], while other contributors include lost income and employment disruption. Specifically, cancer survivors may reduce work hours, change jobs, or permanently exit the workforce during and after treatment [4-7]. Job loss or reduced work capacity decreases household income, amplifying financial stress [4,6,7]. These financial hardships can place survivors at heightened risk for material hardship, including food insecurity [8-13].

Food insecurity is characterized as limited or uncertain access to healthy, sufficient, and nutritious food [14]. In the United States, more than 47 million individuals experience food insecurity at some point during the year [15]. Prevalence among cancer survivors nationally is approximately 10-15%, though some subgroups report higher rates [8-10,12,13]. The adverse effects of food insecurity on health include malnutrition, compromised immune function, reduced treatment adherence, and poorer survival outcomes [14,16,17]. Food insecurity is also linked to diminished health-related quality of life [18]. Data from local and regional studies suggest that employment disruption may place cancer survivors at greater risk of food insecurity, as a Kentucky pilot study found that food-secure survivors were six times more likely to be employed than food-insecure survivors [10]. A national study conducted during COVID-19 did not find a significant association between food insecurity and employment status [11]. However, there is a scarcity of data examining the association between employment status and food insecurity at the national level during non-pandemic times. It is likely that food access is impacted by broader social and household factors.

Marital status, a well-established social determinant of health, plays a role in shaping access to healthy, nutritious foods in the general population [19,20], and it is plausible that it may affect the likelihood of food insecurity among cancer survivors. Married cancer survivors may benefit from pooled financial resources, shared caregiving, and emotional support, all of which reduce vulnerability to material hardship [20,21]. In contrast, unmarried cancer survivors, such as those who are single, divorced, or widowed, may be at risk of greater financial strain, which could possibly place them at risk of experiencing food insecurity [19]. Indeed, unmarried patients with cancer are more likely to experience mortality [22]. However, the potential impact of marital status on food insecurity in cancer survivors is likely larger now than it had been previously due to recent trends in marriage rates. Over the past 50 years, there has been a sharp decline in the number of households with married couples in the United States, now constituting less than half of all households in the country [23]. Despite these emerging trends, few studies have directly examined the effect of both marital status and employment disruption on food insecurity among cancer survivors. The objective of this study was to examine the association between marital status and employment disruption and the risk of food insecurity among a nationally representative sample of cancer survivors in the United States.

## Materials and methods

Study sample

This cross-sectional study used data from the 2016-2017 Medical Expenditure Panel Survey (MEPS), a nationally representative survey administered annually by the Agency for Healthcare Research and Quality. The MEPS collects data on healthcare utilization, insurance, and health status among the non-institutionalized United States population [24]. We used the full-year consolidated files for 2016-2017, which also include the Experiences with Cancer Survivorship Supplement [25]. We then merged these data with the food insecurity files for 2016 and 2017. Adults ≥18 years old who self-reported a history of cancer and provided valid responses to both the cancer supplement and food insecurity dataset were included in the study. Respondents with missing responses on questions related to cancer history or food insecurity were excluded. MEPS data are fully deidentified and in the public domain. This study was approved by the Howard University Office of Regulatory Research Compliance, Institutional Review Board (approval number: IRB 2025-2099; Dated March 3, 2026).

Description of variables

In one segment of the Experiences with Cancer Survivorship Supplement, respondents are able to report their work status at cancer diagnosis [25]. Respondents with no employment at diagnosis were categorized as no employment. Those retaining their employment pattern after diagnosis were classified as retaining full employment. Those with employment at diagnosis who subsequently experienced a disruption such as prolonged time off, a shift in hours, or a change in work schedule were characterized as having employment disrupted. For marital status, respondents reported whether they were married, widowed, divorced, separated, or never married. For analysis purposes, marital status was defined with three groups: currently married, formerly married, or never married. Food insecurity, the primary outcome, was assessed using the United States Department of Agriculture (USDA) Adult Food Security Survey [26]. There are 10 items on this survey, and respondents with a total score ≥3 were classified as having food insecurity.

Several covariates were included. Gender was classified as male or female. Age was grouped as 18-44 years, 45-65 years, or 66 years and over. Race and ethnicity were grouped into non-Hispanic White, Black/African American, Hispanic, or other. Household income used federal poverty level (FPL) designations: <100%, 100-200%, and >200%. Insurance status was reported as any private, public only, or uninsured. Social support was measured using a combined caregiver support variable reflecting whether a spouse only, a child only, both a spouse and a child, or no caregiver provided care, as well as whether any caregiver changed their work schedule to provide care.

Statistical analysis

Descriptive statistics were used to summarize the frequency of observations for categorical variables in the sample before weighting was applied. Survey weights were then applied to account for the complex sampling design of the MEPS and generate nationally representative estimates of proportions. Associations between marital status and study covariates were assessed using design-based F-tests with the Rao-Scott second-order correction (adjusted Wald F statistic). The joint association of marital status and employment status with food insecurity was tested with a design-based adjusted Wald F-test, using a composite variable representing the nine combinations of marital status and employment status. Weighted logistic regression analyses were conducted to examine the relationship between marital status, employment status, and food insecurity while adjusting for relevant covariates. Statistical significance for each regression coefficient was assessed using Wald t-tests within the survey-weighted logistic regression framework. Statistical analyses were conducted using RStudio version 2024.04.02+764 (released 2024; Posit Software, PBC, Boston, Massachusetts, United States). The alpha level was set a priori at 0.05.

## Results

The sociodemographic characteristics of the population are presented in Table 1. The total unweighted sample size was 1,851 respondents, representing over 18 million cancer survivors nationally. Overall, 59.2% were currently married, 33.3% formerly married, and 7.5% never married. Most respondents were over 65 years of age (54.8%), female (55.9%), and non-Hispanic White (83.8%).

**Table 1:** Baseline characteristics and marital status following diagnosis among a national sample of cancer survivors, Medical Expenditure Panel Survey 2016-2017 Values in the second column are unweighted counts with weighted column percentages. Values in the three marital status columns are weighted row percentages and sum to 100% across each row. The total unweighted sample was 1,851 respondents, representing a weighted total of 18,620,227 cancer survivors: currently married, 11,018,203 (59.2%); formerly married, 6,206,378 (33.3%); never married, 1,395,646 (7.5%). Associations between marital status and each characteristic were assessed using Rao-Scott second-order-corrected, design-based F-tests (adjusted Wald F statistic; survey package in R, function svychisq). Percentages may not sum to exactly 100% because of rounding. FPL: federal poverty level

Variable		Unweighted n (weighted %)	Currently married (weighted %)	Formerly married (weighted %)	Never married (weighted %)	Test statistic	p-Value
Age	Young (18 to 44 years)	161 (8.6)	59.1	18.4	22.5	F(4, 265) = 15.67	<0.001
	Middle (45 to 65 years)	650 (36.6)	63.1	27.2	9.7		
	Older (over 65 years)	1,040 (54.8)	56.6	39.8	3.6		
Gender	Female	1,064 (55.9)	52.3	40.1	7.6	F(2, 267) = 17.02	<0.001
	Male	787 (44.1)	67.8	24.8	7.4		
Race	Non-Hispanic White	1,395 (83.8)	61.0	32.6	6.4	F(6, 263) = 4.32	<0.001
	Black/African American	183 (5.7)	38.6	49.2	12.2		
	Hispanic	193 (6.1)	52.0	36.6	11.4		
	Other	80 (4.4)	61.2	22.6	16.2		
Employment status	Retain full employment	652 (38.0)	62.8	30.7	6.5	F(4, 265) = 4.88	<0.001
	Employment disrupted	411 (22.8)	63.5	26.7	9.8		
	No employment	788 (39.2)	53.1	39.7	7.1		
Highest degree earned	Less than high school	308 (12.8)	44.7	45.1	10.2	F(4, 265) = 5.15	<0.001
	High school	774 (41.5)	59.0	34.5	6.4		
	College	764 (45.7)	63.5	28.8	7.8		
Insurance status	Private insurance	1,135 (67.1)	65.9	27.6	6.5	F(4, 265) = 13.40	<0.001
	Public insurance	682 (31.2)	45.6	45.5	8.9		
	Uninsured	34 (1.7)	43.0	35.4	21.6		
Income level	Poor (<100% FPL)	255 (8.6)	29.9	56.8	13.3	F(4, 265) = 22.84	<0.001
	Near poor (100% to 200% FPL)	348 (15.8)	37.5	51.6	10.9		
	Medium to high (>200% FPL)	1,248 (75.6)	67.0	26.8	6.1		
Caregiver support	No caregiver	1,263 (69.4)	54.4	35.5	10.2	F(6, 263) = 40.81	<0.001
	Spouse only	361 (19.3)	85.1	14.3	0.6		
	Child only	115 (5.2)	6.3	88.5	5.2		
	Spouse and child	112 (6.1)	77.1	22.4	0.6		
Caregiver took time off to provide care	Yes	136 (7.4)	67.3	26.7	6.0	F(2, 267) = 1.59	0.206
	No	1,715 (92.6)	58.5	33.9	7.6		

Significant differences across marital status were found for almost all sociodemographic characteristics. Marital status differed significantly by gender (F(2, 267) = 17.02, p < 0.001), as a greater percentage of male respondents were married at the time of interview (67.8%) when compared to female respondents (52.3%). There were also differences in marital status across race (F(6, 263) = 4.32, p < 0.001), as more than 60% of non-Hispanic White respondents were married, compared with approximately 38% of Black/African American and 52% of Hispanic respondents. Differences were likewise observed by employment status (F(4, 265) = 4.88, p < 0.001), with 63.5% of those experiencing work disruption currently married and 26.7% of those who were formerly married. Marital status varied by insurance status as well (F(4, 265) = 13.40, p < 0.001), as the uninsured group had the highest percentage of never-married individuals (21.6%). Income level was also associated with marital status (F(4, 265) = 22.84, p < 0.001). Among respondents living over 200% of the FPL, the majority (67.0%) were currently married, while those with very low incomes (less than 100% of the FPL) had higher percentages of formerly married participants (56.8%) and those who never married (13.3%). Caregiver support was also strongly associated with marital status (F(6, 263) = 40.81, p < 0.001), as a large majority of respondents who received care from a spouse only were currently married (85.1%), whereas those who received care from a child only were most often formerly married (88.5%).

Figure 1 shows the proportion of respondents who reported food insecurity stratified by marital status and employment status. A design-based adjusted Wald F-test with Rao-Scott second-order correction indicated that there was a significant joint association between food insecurity, marital status, and employment status (F(8, 261) = 7.48, p < 0.001). Among those who were married, the prevalence of food insecurity ranged from 1.3% of those who retained full employment to 4.1% for those experiencing employment disruption. Among those who were formerly married, food insecurity was reported by 10.8% of those with no employment, 9.7% of those with disruption to employment, and 6.9% of those retaining full employment. Among those who were never married, food insecurity was reported by 13.5% of those with no employment, 29.2% of those with employment disruption, and 9.4% of those retaining full employment.

**Figure 1:**
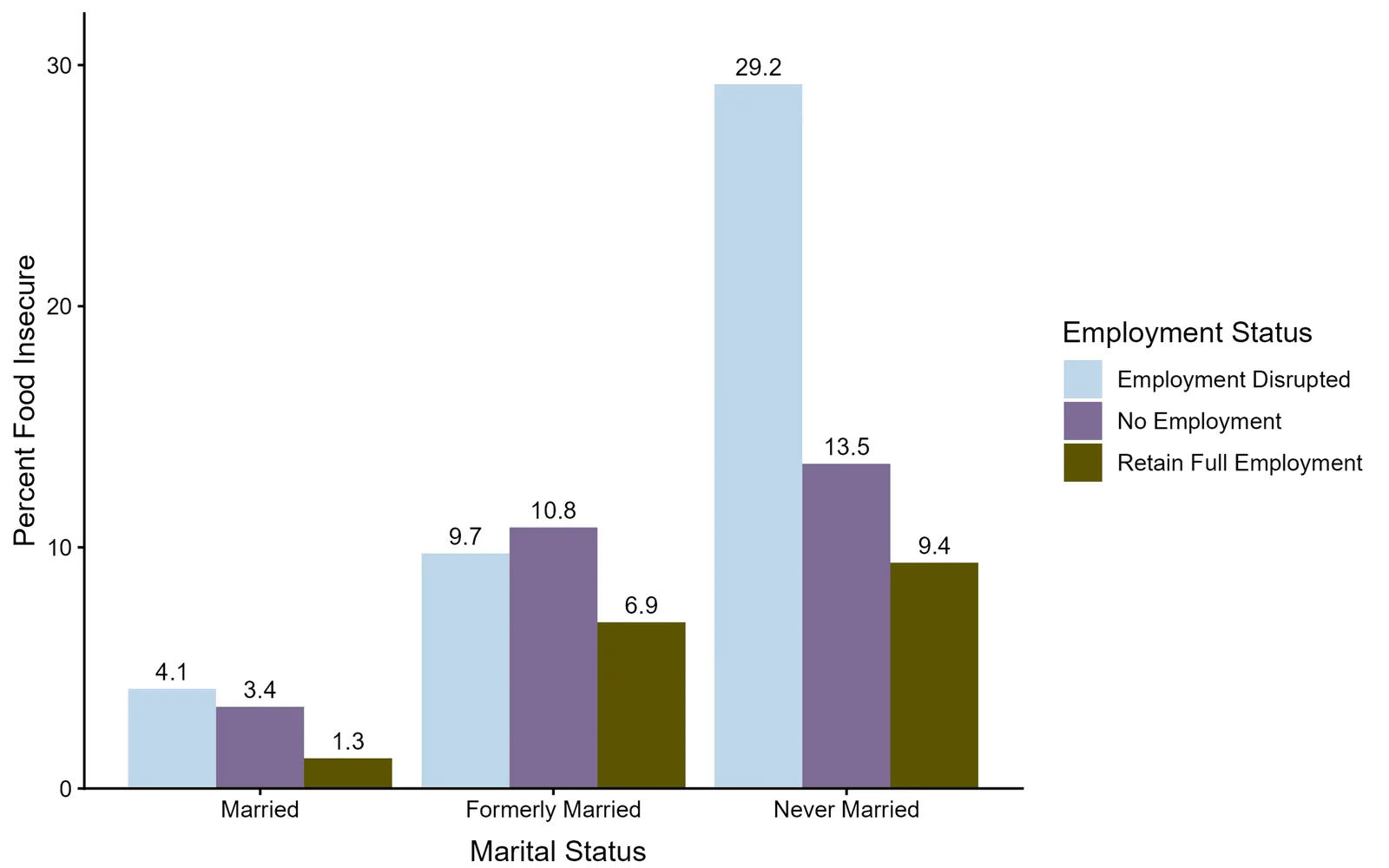
Food insecurity among a national sample of cancer survivors by marital status and employment status Weighted percentage of cancer survivors reporting food insecurity by marital and employment status after diagnosis, Medical Expenditure Panel Survey. Employment 2016-2017 status reflects post-diagnosis work pattern: disrupted, no employment at diagnosis, or retained full employment. Food insecurity was defined as a score of 3 or higher on the Adult Food Security Survey. Differences were significant (F(8, 261) = 7.48, p < 0.001, Rao-Scott second-order-corrected, design-based adjusted Wald F-test). Unweighted n = 1,851.

Table 2 shows the results of the multiple logistic regression analysis. Survivors who experienced employment disruption had 2.46 times the odds of experiencing food insecurity compared with those who retained full employment (AOR = 2.46; p = 0.005). When examining age, younger adults (18-44) had a higher likelihood of experiencing food insecurity compared with middle-aged adults, with an adjusted odds ratio (aOR) of 1.86 (p = 0.049). In contrast, older adults were less likely to experience food insecurity relative to middle-aged adults (aOR = 0.29, p < 0.001). Food insecurity was also more prevalent among never-married survivors (aOR = 2.52, p = 0.014) and formerly married survivors (aOR = 2.14, p = 0.003) compared with married individuals. Respondents in households with incomes above 200% of the federal poverty level had a lower likelihood of experiencing food insecurity compared to those living in households where the income was between 100% and 200% of the federal poverty level (aOR = 0.18, p < 0.001). Black/African American survivors had higher odds of food insecurity compared with non-Hispanic White survivors, yet this association was at the border of statistical significance (aOR = 2.02, p = 0.051). Hispanic survivors did not significantly differ from non-Hispanic White survivors in the odds of food insecurity (aOR = 1.61, p = 0.163). No form of caregiver support was significantly associated with food insecurity in the fully adjusted model (all p > 0.05).

**Table 2:** Weighted multivariable logistic regression of factors associated with food insecurity among cancer survivors, Medical Expenditure Panel Survey 2016-2017 Results are presented as adjusted odds ratios with 95% confidence intervals from a single weighted logistic regression model accounting for the complex survey design of the Medical Expenditure Panel Survey. Statistical significance was defined a priori as p < 0.05. Total unweighted sample n = 1,851. OR: odds ratio; CI: confidence interval; FPL: federal poverty level

Variable		Adjusted OR	95% CI	p-value
Marital status	Currently married	Reference	-	-
	Formerly married	2.14	1.29-3.54	0.003
	Never married	2.52	1.21-5.24	0.014
Employment status	Retain full employment	Reference	-	-
	Employment disrupted	2.46	1.32-4.58	0.005
	No employment	1.37	0.76-2.50	0.297
Age	Young (18 to 44)	1.86	1.00-3.45	0.049
	Middle (45 to 65)	Reference	-	-
	Older (over 65)	0.29	0.15-0.54	<0.001
Gender	Female	1.07	0.67-1.71	0.767
	Male	Reference	-	-
Race	Non-Hispanic White	Reference	-	-
	Black/African American	2.02	1.00-4.10	0.051
	Hispanic	1.61	0.82-3.14	0.163
	Other	0.38	0.09-1.65	0.196
Highest degree earned	Less than high school	1.02	0.55-1.90	0.943
	High school	1.05	0.61-1.81	0.852
	College	Reference	-	-
Insurance status	Private insurance	Reference	-	-
	Public insurance	2.07	1.22-3.52	0.007
	Uninsured	1.76	0.56-5.54	0.332
Income level	Poor (<100% FPL)	1.36	0.80-2.31	0.253
	Near poor (100% to 200% FPL)	Reference	-	-
	Medium to high (>200% FPL)	0.18	0.10-0.33	<0.001
Caregiver support	No caregiver	Reference	-	-
	Spouse only	0.59	0.33-1.08	0.085
	Child only	0.83	0.38-1.80	0.630
	Spouse and child	0.71	0.30-1.69	0.438
Caregiver took time off to provide care	No	Reference	-	-
	Yes	1.68	0.77-3.67	0.189

## Discussion

Being unmarried was associated with a higher likelihood of food insecurity in this nationally representative sample of cancer survivors. Similar to previous research [20,27], these findings suggest that survivors without the social, emotional, and financial support of a spouse may experience greater material hardship. Family as well as other informal networks promote adherence to pharmacotherapy and assist with transportation, meal preparation, and financial management that help shield survivors from hardship [27]. In addition, being married provides some level of protection from financial toxicity through pooled incomes and shared expenses within the household [20]. This impact may be larger now than previously seen, as over the past 50 years, married couples have come to constitute less than half of all households in the United States, meaning future survivors may be less likely to benefit from this informal support and economic buffering [23].

We also examined factors associated with marital status. As age increased, the proportion of never-married survivors decreased, while the proportion of formerly married survivors increased. Men were more likely to be married than women, and Black and Hispanic survivors were less likely to be married than non-Hispanic White survivors. Importantly, racial and ethnic differences in marital status should not necessarily be interpreted as differences in access to caregiving support. A study by Borsky et al. indicates that cancer survivors of a race/ethnicity other than White were more likely than non-Hispanic White survivors to report having an informal caregiver [21], suggesting that support may also be provided through broader family and social networks. Qualitative research among married African American breast cancer survivors also found variability in the emotional and tangible support provided by spouses [28], suggesting that marital status alone may not fully capture the availability or adequacy of caregiving support in this demographic. Married survivors in the current study had higher educational attainment than those who were formerly or never married, and survivors with lower income (below 200% of the FPL) had higher proportions of formerly and never-married individuals, whereas those with higher income (over 200% of the FPL) were more likely to be currently married.

Employment disruption was also associated with food insecurity in this national sample. Chemotherapy can impair a patient's ability to work through fatigue, cognitive impairments, and treatment time, reducing work hours or causing patients to leave work entirely [5]. Previous work shows that employment disruption is more likely to lead to material and economic hardship among cancer survivors who are younger, ethnic minorities, have lower incomes, and reside with dependents [6]. Consequently, cancer survivors who are more likely to be unmarried and those who experience the greatest distress from employment disruption share many characteristics. In the current study, nearly 30% of never-married cancer survivors who experienced employment disruption also experienced food insecurity.

Previous studies suggest that food insecurity among cancer survivors experiencing financial hardship may be addressed through clinical, community, and workplace-based interventions. A randomized trial involving cancer patients being treated in safety net settings demonstrated that food pantries, vouchers, and grocery delivery helped to alleviate food insecurity [29]. The food vouchers combined with the pantry in particular yielded positive benefits [29]. Financial navigation programs also have the potential to benefit cancer survivors by mitigating financial toxicity, yet their results are mixed [30]. Therefore, they are likely best used within a multifactorial approach. A recent review of randomized trials indicated that multiple types of intervention, such as vocational counselling, patient education, and exercise, may facilitate cancer survivors returning to work [31]. Altogether, these findings lend support for pairing screening and referral for food and financial needs with individualized employment and rehabilitation support, particularly for unmarried survivors or those with limited household support [32].

These results also have important implications for broader policy approaches. Although the federal Family and Medical Leave Act confers job protection during some periods of illness, it does not guarantee paid leave, limiting its usefulness for many vulnerable survivors [33]. In contrast, paid leave policies have been associated with improved economic stability and positive health outcomes [34], suggesting that extending paid leave could help survivors maintain employment and reduce material hardship. This may be especially beneficial for survivors with lower socioeconomic status and those who are unmarried.

Strengths and limitations

This study has strengths that should be noted. The use of a large, nationally representative sample helps to enhance external validity. Food insecurity was measured using a validated scale, and both marital and employment status were characterized using multiple categories. The inclusion of caregiver support and other sociodemographic factors allowed us to conduct a more comprehensive examination of the social and economic circumstances associated with food insecurity among cancer survivors. This study used the 2016-2017 MEPS panels, the most recent available, with the Experiences with Cancer Survivorship Supplement. Although economic conditions and healthcare policy have since evolved, the underlying determinants of employment disruption, financial hardship, and food insecurity among cancer survivors remain relevant.

This study also has some limitations. First, the cross-sectional design precludes establishing causal relationships between food insecurity, employment disruption, and marital status. Food insecurity was self-reported and may be subject to recall and social desirability bias, leading to either underreporting or overreporting. We were also unable to include clinical details such as cancer stage, treatment type, or time since diagnosis, which could influence both employment disruption and food insecurity. Despite these limitations, the large nationally representative sample strengthens the generalizability of these findings.

## Conclusions

In this study featuring a nationally representative sample of cancer survivors, marital status and employment disruption were both associated with the likelihood of food insecurity. Specifically, those who had never been married and survivors who experienced some type of change in their employment had the highest risk of food insecurity. These associations persisted even after adjusting for several sociodemographic variables, which suggests that marital status and employment circumstances influence the likelihood of experiencing food insecurity beyond socioeconomic status alone. Accordingly, optimal care of cancer survivors should extend beyond clinical management alone and include strategies to identify survivors who may benefit the most from interventions to help them deal with the financial toxicity and material hardship that accompany a cancer diagnosis. Specifically, cancer survivors who are unmarried or have a change in their employment status may benefit from food and nutrition programs. Further research should evaluate programs that protect income for cancer survivors, such as paid family and medical leave. Given the cross-sectional design of this study, future studies should incorporate a longitudinal design to elucidate the temporal relationships among employment disruption, marital status, and food insecurity.
